# Girls Just Wanna Have Fun: a process evaluation of a female youth-driven physical activity-based life skills program

**DOI:** 10.1186/2193-1801-3-401

**Published:** 2014-08-03

**Authors:** Corliss N Bean, Tanya Forneris, Tanya Halsall

**Affiliations:** School of Human Kinetics, University of Ottawa, 125 University Pvt 406B, Ottawa, ON Canada

**Keywords:** Positive youth development, Physical activity, Process evaluation, Community programming, Female youth

## Abstract

**Introduction:**

Integrating a positive youth development framework into physical activity programming has become popular as it is believed that this integration can create the development of both physical and psychosocial skills. However, there has been a lack of intervention fidelity research within the field of positive youth development.

**Case description:**

The Girls Just Wanna Have Fun program was designed in response to increased calls for physical activity programs for female youth and is a theoretically-grounded physical activity-based life skills program that aims to empower female youth. The purpose of this paper was to provide a detailed description of the program and a process evaluation of the first year of program implementation. From interviews with youth and leaders, as well as documentation from the leaders’ weekly online log of each implemented session, themes emerged regarding the successes.

**Discussion and evaluation:**

Findings from this study indicated that program goals were attained and it appears that the program was implemented, for the most part, as designed. The themes related to successes included using activities to facilitate relational time, providing intentional opportunities for leadership, having communicative program leaders who supported one another, and engaging youth in different types of physical activity. The themes related to challenges included difficulties with facility and transportation, some activities being too much like schoolwork, and social distractions and cliques. Included in the paper is a discussion of practical implications and recommendations for community programmers, as well as future directions for the program.

**Conclusions:**

Overall, this process evaluation represents an important step in responding to calls for increased evaluation in community-based programs and aids in understanding the process in which positive youth development programs can be effectively implemented.

The theoretical perspective of positive youth development (PYD) sees youth in terms of their potential for healthy development (Catalano et al. 2002) and can be defined as the “development of personal skills or assets, including cognitive, social, emotional, and intellectual qualities necessary for youth to become successfully functioning members of society” (Weiss and Wiese-Bjornstal 2009, p. 1). By providing youth with opportunities to facilitate these qualities, youth can acquire positive life skills, such as goal-setting, time-management, respect, self-regulation, communication and problem solving that not only helps to facilitate youth’s successful transition to adulthood, but also enables them to lead meaningful lives and contribute positively in society (Catalano et al. 2002; Theokas et al. 2005). As a result, youth development programs aim to create opportunities to foster the health and well-being of youth (Theokas et al. 2005). Integrating a PYD framework into the physical activity (PA) and sport domains has become popular, as it is also believed that this integration can create a dually productive environment: the development of both physical *and* psychosocial skills (Danish et al. 2004; Gould and Carson 2008).

Systematic reviews of PA interventions for youth support that PA interventions directed at youth can be effective (Dobbins et al. 2009; Thomas et al. 2004). Stone et al. (1998) reported that the majority of such interventions were hindered by poor design and were not community-based. More specifically, recommendations for future research by Stone and colleagues included more studies that focus on increasing PA in out-of-school contexts, process evaluation frameworks, and the inclusion of program descriptions within the studies themselves. There is a growing need to address youth PA in the community as, to date, the knowledge translation from youth PA research to practice in the community is weak (Kelly et al. 2012; Peters et al. 2013). Moreover, the Active Healthy Kids Canada (2011) report card identified that community-based programming is needed as youth are highly inactive between 3 and 6 PM and less than fifty percent of community-based programming have PA as a primary component, or target youth.

Coakley (2011) emphasized that the development of PYD programs designed to enhance the agency of youth can be challenging and therefore advises that community programmers form cooperative and mutually supportive relationships with scholars in order to implement and test the effectiveness of such programs. This research represents such a relationship between researchers at a University and the Boys and Girls Club (BGC), both located in Eastern Ontario, Canada. Together, these researchers and the local BGC developed and implemented the Girls Just Wanna Have Fun (GJWHF) program, a PA program that incorporated a PYD approach through the teaching of life skills.

GJWHF was developed in response to an identified gap in PA programming for female youth. From 2008–2009, it was identified that only 243 females compared to 1012 males were participating in sport and PA programs across several BGC locations within the city. This issue is not unique to BGCs within Canada, as the BGC of America designed a similar program called ‘Smart Girls’, to address gender equity (Boys and Girls Club of America 2013). Because of such discrepancies, there is a need for more PA and sport programs for female youth. Moreover, a pilot study was conducted with youth from the local BGC prior to the development of GJWHF at the local BGC. Findings indicated that female youth wanted more opportunities to be active in a girls’-only environment that fostered social support (Forneris et al., 2013).

In regards to specific PA programming for female youth, it is recommended to incorporate psychosocial skill instruction and experiences that aim to enhance self-esteem, positive body image, positive attitudes towards PA, and motivation to participate in PA (DeBate et al. 2009; Futris et al. 2013; Rauscher et al. 2013). Programs should aim to also provide social environments that offer a variety of activities that girls consider enjoyable (Martin et al. 2009). For example, Cooky (2009) argued that the environment in which female participants engage in a program needs to reflect their culture and norms, and in doing so enables participants’ interest in the program She recommended that when considering both the organization and implementation of girls’ recreation sport programs, both female youth participants and adult organizers should co-construct the interest in sport, as this can be used as an empowerment tool for girls (Cooky, 2009).

A number of female-only PA programs have been developed in North America. One program entitled Sporting Chance, offered by Girls Incorporated, aims to expose female youth to a number of sports and potential career options, build participants’ athletic skills, and enhance knowledge of health (Girls Incorporated 2013). Another program, Girls on the Run, is a PA-based program that incorporates running and life skills. Participants meet with trained coaches afterschool two times per week for 12 weeks to participate in life skills and running activities. The program concludes with the youth completing a 5 km run event. This program has been evaluated by a number of scholars and findings have indicated that youth perceived the program to help with social self-concept, physical self-concept, and attitudes towards healthy living (DeBate et al. 2009; Martin et al. 2009; Waldron 2007). The youth also discussed that social support received from coaches and teammates was significant and important in facilitating the previously stated outcomes (Martin et al. 2009). In study conducted by Wright et al. (2008) that utilized the Teaching Personal and Social Responsibility (TPSR) model in a program for female youth, the authors indicated a key to utilizing this model successfully, is the ability to establish relevance for participants; making the activities within the program relevant to the participants. In another study, Wright et al. (2012) illustrated the use of lessons learned in adapting a PA-based program for girls that was based on the TPSR model. The authors noted the importance of providing flexible leadership and engaging the girls in the direction of the program.

Although there has been an increase in female-only programs that integrate PA and a PYD approach, there has been relatively little evaluation, particularly process evaluations, of such programs conducted (Whitley et al. 2014). Although conducting outcome evaluations aiming to understand the impact of such programs are important, process evaluations are equally important as they enable researchers and practitioners to gain insight into best practices for these programs (Linnan and Steckler 2002). Linnan and Steckler (2002) explained that an important piece in ensuring that interventions can be improved and sustained over time, is understanding the program components that enable the program to be effective and to understand under what conditions a program or intervention may be effective. Therefore, in order to advance science in the field of PA and youth development, it is important to learn about the successes and challenges of programs designed to positively impact the development of youth.

Windsor et al. (1984) described process evaluation as:

Part of a formative evaluation and assesses whether specific elements such as facilities, staff, space, or services are being provided or being established according to the given program plan…Process evaluation involves documentation and description of specific program activities, monitoring the frequency of participation by the target population and is used to confirm the frequency and extent of implementation of selected programs or program elements. (p. 3)

Similarly, Durlak and Dupre (2008) asserted that interventions cannot be adequately tested to determine effectiveness without a focus on the implementation of the program. They discussed the importance of examining what aspects of the program were delivered and how well they were conducted as essential for understanding the validity of interventions. In addition, Hodge et al. (2013) argued that there is a dearth of literature that provides the reader with a detailed description of the intervention, the actual content within each program session, and how the program is implemented which is important in providing high quality youth programs. In sum, the collection of process data and provision of a detailed program description is an essential part of program evaluation and is needed to enhance the quality of youth programming.

Moreover, there is a need for research examining the realities of youth programs at a practical level. All too often only the positive outcomes of a program are reported and the reality of the day-to-day implementation is neglected. A process evaluation can help understand how a program is delivered as well as how it is experienced by both the program leaders and the youth who receive the program. Therefore, the purpose of this paper was to provide a detailed description of the GJWHF program and to also present the results of the process evaluation. This study used a qualitative approach by examining the GJWHF program implementation logbook and conducting semi-structured interviews, methods that are often employed in conducting process evaluations (Linnan and Steckler 2002). Three research questions were proposed: 1) How well was the program implemented as designed?; 2) What were the perceived successes of the program?; and 3) What were the challenges faced in implementing the program?

## Method

### Context – a detailed description of the GJWHF program

The GJWHF program had three main program goals: 1) to provide female youth with opportunities to be physically active, 2) to facilitate life skill development, and 3) to enable opportunities for youth voice. In order to achieve these three goals, the GJWHF program was based primarily on and Hellison’s (1995, 2011) TPSR model with the incorporation on life skills activities from Danish et al. (2004) Sports United to Promote Education and Recreation (SUPER) program model. These program frameworks are considered complimentary as they both utilize explicit life skill programming to foster youth development (Holt, 2008). The rationale of using the TPSR model as the primary framework was because a key value in this model is providing the youth with voice, particularly with regard to the types of sports and PA they wanted to participate in (see details below in Table 1).

**Table 1:** **Summary of GJWHF program sessions including date, attendance rate, and what life skill and sport or physical activity was of focus**

Session #	Date	Attendance	Life skill	Sport/Physical activity
1	Sept. 27	10	Communication	Co-operative games
2	Oct. 4	10	Teamwork	Co-operative games
3	Oct. 11	7	Confidence & courage	Co-operative games
4	Oct. 18	11	Confidence & courage	Co-operative games
5	Oct. 25	10	Respecting others	Kickboxing
6	Nov. 1	10	Respecting others	Kickboxing
7	Nov. 8	12	Responsibility	Lacrosse
8	Nov. 15	12	Responsibility	Lacrosse
9	Nov. 22	11	Dare to dream	Volleyball
10	Nov. 29	12	None—(taxis late)	Basketball
11	Dec. 6	14	Dare to Dream	Dance
12	Dec. 13	10	None (holiday party)	Dance
13	Jan. 10	10	Goal setting	Basketball
14	Jan. 17	0	None (cancelled bad weather)	None (cancelled bad weather)
15	Jan. 24	6	Overcoming obstacles	Co-operative games
16	Jan. 31	12	Seeking help from others	Co-operative games
17	Feb. 7	14	Seeking help from others	Co-operative games
18	Feb. 14	10	None (skating)	Skating
19	Feb. 21	13	Self-talk & thought control	Yoga
20	Feb. 28	12	Relaxation	Yoga
21	Mar. 6	14	Focus	Yoga
22	Mar. 13	5	Empowerment	Walk
23	Mar. 20	13	Hygiene	Walk
24	Mar. 28	10	None (swimming)	Swimming
25	Apr. 4	8	None (swimming)	Swimming
26	Apr. 10	9	Empowerment & Confidence	None (photos)
27	Apr. 17	9	Leadership	Co-operative games
28	Apr. 24	7	Appreciating differences & youth planning	Co-operative games
29	May 1	8	Youth planning	Co-operative games
30	May 8	10	Youth implementing	Co-operative games
31	May 15	13	None (end of program party)	Walk

The TPSR model was developed from work with at-risk youth in after-school sport and physical education programming in schools. This model focuses on developing a strong instructor-participant relationship that allows for the gradual empowerment of youth (Hellison et al. 2008). The five main levels of the TPSR model include: (1) Personal Responsibility or Self-Control; (2) Effort; (3) Self-Coaching; (4) Leadership; and (5) Transference. Each of these five components arose as practical programming guidelines that have been shown to lead to positive outcomes for youth (Hellison and Walsh 2002; Martinek et al. 2006; Walsh et al. 2010). Personal responsibility or self-control refers to the ability to control one’s behavior and conduct, effort refers to participants’ ability to apply themselves to a given task, self-coaching refers to the ability to improve in a chosen area using goal setting and planned practice, leadership refers to the ability to direct a group towards an agreed upon goal, and transference refers to the ability to use the skills outlined above in contexts outside of the program (e.g., school, home, etc.) (Hellison and Walsh 2002; Martinek et al. 2001). Within each session, the youth were reminded of the five components (e.g., taking responsibility for their actions) and as the program progressed the leaders included activities and opportunities to allow the youth to increase personal responsibility, effort, the ability to self-coach and take on greater leadership roles. The youth were also encouraged to transfer the life skills being taught in the program outside of the program.

To help achieve these objectives, the TPSR model uses a specific program structure (see Table 2 for detailed program structure). The first 5–10 minutes of the session is set aside for relational time; a time where leaders check in with the youth to see how things are going in their lives. Within the GJWHF program, the ‘Rose and Thorn’ activity was used during the relational time which involved breaking into small groups and then each youth shared one rose (something positive that happened that week) and one thorn (a challenge that happened that week). The relational time was then followed by an awareness talk. The awareness talk (20–25 minutes) focused on the different TPSR levels and a variety of life skills including goal setting, confidence and courage, respecting others, and seeking help from others. While Hellison (2011) noted that the awareness talk can be kept fairly short, he also noted the importance of flexibility when implementing the TPSR model. When designing the program, it was decided to lengthen this section of the sessions as it was deemed important that in addition to talking about the skills, it was critical to have the youth engage in activities to specifically practice the skills. This rationale is supported by the work of several researchers (Danish 1997; Gould and Carson 2008; Petitpas et al. 2005) who emphasized that youth learn best by doing, highlighting the importance of providing youth the opportunity to practice skills. In addition, program material from SUPER was integrated in the awareness talk, as in previous literature there is a lack of specific details or activities for this portion of a TPSR program. As a result, workshop activities from SUPER were integrated to help leaders have resources for teaching the levels. For example, when working with youth on effort specific activities from the goal setting workshops in SUPER were implemented. The awareness talk was followed by the PA plan (20–25 minutes) which was time that the youth engaged in a sport or form of PA. At the beginning of the program, cooperative games were incorporated to help the youth get to know each other and to provide them time to choose the physical activities that they wanted to incorporate into the program. Once the leaders had a list of different physical activities from the youth, the participants were asked every two weeks what activity they wanted to engage in next. Finally, a group debrief (5–10 minutes) occurred at the conclusion of each session where the leaders and youth discussed progress that had been made in the program session that day, as well as challenges or difficulties faced.

**Table 2:** **Program structure for each session**

Component	Description
Relational time	The Rose and Thorn activity (5–10 min), where each of the youth share one experience that went well for them during the week and one that was a challenge; used to strengthen relationships between youth and leaders as well as among youth themselves
Awareness talk	The Awareness talk was used to teach a variety of life skills included the five levels of responsibility (self-control, effort, self-coaching, leadership, transfer) as well as other life skills (respect, positive self-talk, goal setting).
Physical activity/Sport	The youth were provided with choice as to what type of activities they wanted to participate in. Some of the activities the youth chose included: basketball, volleyball, swimming, skating, dance, etc. The life skill of the session was integrated into the activity.
Group discussion	At the end of every session a debrief took place with the leaders and youth to discuss progress and challenges of the session. This is also when youth completed their self-evaluations.

Throughout the program, there were a number of sessions when a unique structure was implemented. For example, when skating and swimming took place, the group had to travel to an outside location and there was not enough time for a 25 minute awareness talk relating to a specific life skill. However, the relational portion of the session (‘Rose and Thorn’ activity) always occurred. Additionally, there was one session where a photographer was brought in to take the girls’ pictures to help them develop a portfolio of themselves to increase confidence and empower the girls. In this session, no specific sport or PA was integrated because of this unique opportunity. In addition, a session was conducted on hygiene as the program leaders thought this was a critical element to discuss with the female youth between the ages of 11 and 14 as they were beginning to go through puberty and to experience changes to their body. Finally, the last three weeks of the program were designed around leadership. The girls were responsible for planning, developing, and implementing PA games and life skill activities to younger youth within the BGC. The girls worked in groups of two or three and had two weeks of planning within their small groups. The following session involved the girls presenting and teaching their younger peers (6 to 9 years old) at the BGC what they had learned from the program.

### Participants & procedure

#### GJWHF program participants

From September 2011 to May 2012, female youth ages 11 to 14 (M =11.75, SD = 1.19) from two BGC locations met at one clubhouse once per week for a 75 minute program. The BGC decided to open the program to female youth from two different clubhouses. The girls were from low income families in a major city in Eastern Ontario. In addition, as this was the first year that the GJWHF program was run, all female youth were participating for the first time; however, their length of involvement at the BGC ranged from two months to nine years.

The program took place at one clubhouse and free transportation was provided to and from the program for youth from the second clubhouse. Either a BGC bus or taxi picked the youth up at their home clubhouse and then dropped them back to their home clubhouse after the program ended for the night. The average attendance rate was 10.4 youth per session; however, this number did fluctuate from five youth (during March break) to 14 youth (see Table 1 for specific attendance rates). Youth did drop out over the course of the program due to competing program alternatives within the clubhouse or lack of interest. However, a total of 10 youth consistently attended the program throughout the year (attending more than 75% of the program sessions) and were involved in this study. All of the youth who participated in the program, regardless of participation rates, were invited to partake in the research and were provided with parental consent and youth assent forms to complete. However, only the 10 youth who did participate consistently in GJWHF agreed to participate in the research and also obtained parental consent. However, it should be noted that participation in the research was not required for participation in the program and it was clearly stated on the consent and assent forms that the participation in the research was voluntary. The parental consent forms were provided at the same time as the program registration forms to provide ample opportunity to collect them from the youth prior to any data collection. The youth assent forms were distributed prior to data collection. All procedures were approved by the University’s Office of Research Ethics and Integrity.

#### Program leadership

Five female staff ran the GJWHF program. From these staff members, two of the leaders were BGC program staff and three were students at a local university. All of the leaders ranged in age from 21 to 46 years old (M = 28.6, SD = 8.94) and all had completed or were in the midst of attaining a degree in the fields of Human Kinetics or Social Work. The primary leader, who is the first author and aided in program development and implementation, was a graduate student at the local university. The leaders outside of the BGC had less experience working with youth and therefore were required to complete the standardized volunteer training at the BGC. Furthermore, all leaders took part in a one day training program that was run by a professor with expertise in the field of program implementation and youth development before the start of the program. Training consisted of outlining the progression of the program by going over the manual and addressing how each session would be structured, based on the TPSR model. The leaders also met 30 minutes before the start of each program session to discuss the plan for the session and at the end of each session a 15 minute debriefing period was held to discuss what went well, what could be improved, and plans for the following week. In addition, the primary leader was responsible for sending out weekly emails of session plans and facilitating the session debrief with the co-leaders post-session. All five leaders participated in this study.

One critical component of this program was the large leader support, as on any given night of the program, there was a minimum of four leaders present, and on most evenings all five leaders were present.

### Measures

#### Leader logbook

Typically, a process evaluation measures fidelity of program implementation (e.g., number of sessions implemented, sessions implemented as designed, leader and participant attendance) and participants’ receptivity and enjoyment of the program (Nakkash et al. 2012; Saunders et al. 2005; Shek and Sun 2012). To document this information, an Leader Logbook entry was completed by all of the leaders and inputted into the GJWHF Leader Logbook by one leader using a web-based survey development tool, Survey Monkey, after the leaders’ debrief meeting. Each entry consisted of the same 10 questions regarding the implementation (date of session; how many youth participated; what life skill was incorporated; were the life skill activities successfully implemented, why or why not?; what PA/sport was incorporated?; were the physical activities successfully implemented, why or why not?; did the session go as planned, if no explain; what went well in the session; what challenges did you face and what could be improved?) and one question that involved rating the group of youth in general on four of the TPSR components (self-control, effort, self-coaching, leadership) on a scale from “Needs Work” (1) to “Great” (4). Transference was not included as it was not possible for the leaders to observe transference. These questions were discussed verbally in the leader debrief session prior to the completion of the Leader Logbook and the same leader recorded the leaders’ comments that were expressed during the debrief session and was also responsible for entering the data online.

#### Semi-structured interviews

In addition, at the end of the program 15 interviews were conducted (10 participants, five leaders). Due to the time constraints of the program, the youth were interviewed at their home clubhouse within one week of the conclusion of the program. As mentioned above, some of the youth dropped out of the program. Although it was planned to interview the youth who had stayed in the program as well as those who dropped out, arranging interviews with the youth who had dropped out proved to be very difficult. Therefore only the 10 youth that participated for the entire program were interviewed. The leaders were also interviewed outside of program time at a place and time convenient for them. Two separate interview guides were developed, one for youth and one for program leaders. The interview guide for the youth was designed to understand their experiences in the program as well as their perceptions of how participation in the program may have impacted their personal development. Questions included: ‘What did you like and not like about the program?’; ‘What did you learn in the program?; ‘What was your experience like working with the program leaders?’; ‘What did you learn by being involved in the program?’; ‘What do you believe has impacted you the most during this program?’; ‘Is there anything you hoped would have happened in or after the program that did not happen?’. The program leader interview guide was designed to understand their experiences implementing the program and their perceptions of program impact. Questions included: ‘How was the GJWHF program perceived by the youth?’; ‘What successes and challenges did you experience related to implementing the GJWHF program?’; ‘What strategies did you use to keep the youth engaged in GJWHF? Which strategies were the most effective?’; ‘What suggestions do you have for improving the GJWHF program club?’. Probes were also used to explore areas of the participants’ experiences further. All of the interviews were recorded using an audio-recorder. The youth interviews ranged from 15 to 42 minutes, while the leader interviews ranged from 25 to 45 minutes.

The youth interviews were conducted by the lead researcher who was also a program leader. It is recognized that having the researcher also be a leader in the program could introduce bias; however, several studies within the field of youth programming have used this approach in the past (Hellison and Walsh 2002; Ward and Parker 2013). Heath et al. (2009) also asserted that youth are often more likely to open up to an individual with whom they know, have interacted with and trust, rather than a third party interviewer. The program leader interviews were conducted by a graduate student with experience in qualitative interviewing who was not involved in the GJWHF program. In addition, in order to further negate the potential for social desirability from the youth, an opportunity was given to the youth to provide feedback anonymously. The youth were given a blank piece of paper in which they could write anything they liked or disliked about the program. Upon completion of this form, the youth placed the paper in a sealed envelope.

### Data analysis

Each interview was transcribed verbatim, resulting in 154 pages of transcripts (12-point font, single spaced). The interview transcripts and the GJWHF online logbook were analyzed using an inductive thematic analysis (Braun and Clarke 2006). Braun and Clarke (2006) argued that using a thematic analysis allows for flexibility when analyzing the data as it allows for the triangulation of different participants’ perceptions. Once all of the interviews were transcribed, the transcripts were read multiple times. Trustworthiness of the data was assured through a collaborative approach to analysis (Creswell 2013). Two investigators analyzed both the transcripts and logbook within the first round of analysis. Each investigator completed the following steps: first, the data were read to become familiar with the data. Upon reading through the data a second time, notes were made in relation to emerging themes. Third, the transcripts and logbook entries were re-read to group responses into broader themes. Finally, the broad themes were organized and relevant quotations identified that supported the emerging themes. Once this analysis process was completed, the investigators met and discussed the established themes.

A number of efforts were made to increase the credibility and trustworthiness throughout the research. First, prior to the start of data collection, the first author participated in a bracketing interview (Finlay 2014; Pollio et al. 1997) related to PYD and PA programming for youth. Second, prior to conducting interviews, participants were reminded that the study was voluntary and of their rights to confidentiality and anonymity. Third, triangulation was also employed within this study. Multiple sources (youth, leaders), as well as multiple methods (logbook, interviews) were used to collect data. The convergence of evidence between multiple sources and methods provides an in-depth, accurate and convincing account than solely one source or one method (Maxwell, 2005). Lastly, as mentioned, trustworthiness of the data was ensured through a collaborative approach to analysis (Creswell, 2013). Investigator triangulation (Yin, 2009) was utilized as all qualitative interview transcripts were analyzed separately by two investigators within the first round of analysis. Discrepancies between the researchers (e.g., how the themes should be labelled and the placement of quotes under different themes as sometimes one quote could support multiple themes) were discussed until an agreement was reached. This process helped to ensure identified themes were consistent with the data collected and helped to verify that themes and sub-themes were accurately represented. Data were then shared with a third investigator, an independent auditor, who was not involved in the GJWHF or the data collection, but was a graduate student who had experience in qualitative data analysis. This investigator examined whether the identified themes were consistent with the data collected and verified that themes and categories were accurately represented, which helped to provide additional validity.

Identification codes were created for each quotation by assigning a number to each participant in the order in which they were interviewed to ensure anonymity and confidentiality. Quotations taken from the Leader Logbook were identified by the date of the session. In addition, for the anonymous feedback provided by the youth, numbers were written on the paper in the order in which they were completed (Y = youth participant; L = leader; LL = Leader Logbook; AYF = anonymous youth participant feedback). For example, the identification code L-4 would indicate that the individual was a leader and was interviewed fourth.

## Results

The first section presents the results focused on fidelity of implementation which was documented based on the analysis of the Leader Logbook. This is followed by the results of the thematic analysis which revealed two overarching themes: successes and challenges. These two themes were comprised of seven sub-themes.

### Fidelity of program implementation

As noted in Table 1, 30 out of 31 planned sessions were carried out (97%). One session was cancelled due to bad weather during the winter months (3%). Twenty-three sessions out of the 30 implemented sessions (77%) were executed using the TPSR structure on which the program was based. Of the seven sessions in which adaptations were made to the typical TPSR program structure, adaptations were preplanned for six of the sessions due to either the time needed for the planned PA or life skill activities (e.g., when the youth went skating, swimming or had the photographer attend) or for holiday and end of session parties. One session was adapted because of issues with transportation being late and therefore no life skill was carried out for that session. Of the remaining 23 sessions, all planned PA and life skill of focus were delivered appropriately. Further, it was noted that youth voice was provided over the course of the program, particularly with regards to the types of PA incorporated in the program. For example, one youth stated: “I liked when you guys would ask us before you just picked any random sport…I liked having a say” (Y-6), while another youth went on to say:

They give you the option of if you wanted to play first or have snacks or do the lesson. I think it was nice because they’d give you the choice of what you feel comfortable doing and they’d make you feel more comfortable and give you more expression. (Y-5)

Lastly, one youth discussed that having a program that was youth-driven was ideal compared to other programs:

I liked how it was sort of our own thing. It was run by the leaders, but we would pitch ideas and [the leaders] would try to do them. With other groups I’ve been in, it’s sort of like they do all the planning; they pitch the ideas and we do them. I like this part. (Y-3)

Therefore, it appears that GJWHF was implemented as designed which may help explain the successes outlined below.

### Successes

Four themes emerged related to program successes which included using ‘Rose and Thorn’ to facilitate relational time, providing intentional opportunities for leadership, having communicative program leaders who supported one another, and engaging youth in different types of physical activity. These themes are highlighted in greater detail below.

#### Using ‘Rose and Thorn’ to facilitate relational time

The importance of using an activity like ‘Rose and Thorn’ was recognized by both youth and leaders as having a positive impact and an effective means of connecting with each other. Originally, the ‘Rose and Thorn’ activity was planned to be an ice breaker at the start of the program, but it was soon understood that the youth enjoyed this time to share with each other. As a result, the ‘Rose and Thorn’ activity was implemented at the beginning of every session during relational time. This activity allowed time for the girls to talk with each other, listen to each other and relate to each other, as well as facilitate a relationship with the leaders. As one leader explained:

Now they [the youth] all come in and it’s like ‘I have a good rose today’, or ‘I have three roses’ and it’s something that they definitely look forward to and they know it’s coming at the beginning—they’ve come to expect it…so we’ve kept it going all year, and I guess it gives the kids some time to reflect on how their week’s been going or some of the problems they’ve been having at school. And they’ll chat about it with you, so it’s good to kind of talk through things with them and then it kind of sets the tone for the rest of the session too. So that’s been a really cool highlight. (L-5)

The youth also felt supported by their peers and leaders in this activity as highlighted by these two quotations: “I liked the ‘Roses and Thorns’. You get to tell what happened, like you can share with everyone what happened in your life. And it kind of helped…like if you don’t have anyone to talk to” (Y-9) and:

In ‘Rose and Thorns’ we get to speak out and say we have a problem, we get to tell people the problem and they’d actually listen…I like ‘Rose and Thorn’ because you got to share what’s going on in your week. I felt like other people would understand what was happening, and what I did and stuff. Like say I was mad at something, they would understand, and not bombard me. (Y-4)

Similarly, the youth felt that this activity helped increase their relationships with the leaders: “I think the ‘Rose and Thorn’ activity is nice—it makes me feel as if they [the leaders] want to know what’s going on and it makes me actually feel more comfortable with them” (Y-5).

Some youth shared that the ‘Rose and Thorn’ activity also helped them to increase their confidence throughout the year: “I can speak out louder in front of them and show my ‘Rose and Thorn’ now. At the first day, I couldn’t do it because I was too shy. Now I’m comfortable sharing in front of everyone” (Y-8). In addition, the leaders discussed that the youth respected and listened to their peers, problem solved, and appreciated each other. Two leaders discussed the importance of having decided as a group to keep the ‘Rose and Thorn’ activity as part of the relational time at the beginning of every session: “That’s what the girls really enjoy and in and of itself – learning to listen to each other, appreciating each other, contributing to the group – that’s what ‘Rose and Thorn’ does and those are life skills too” (L-2) and:

I think it is important, and we don’t want to downplay that. Originally, it was used as an ice-breaker, but the youth definitely get a lot of skills out of it like communication skills, listening to others, respecting others when they’re talking, problem solving, someone’s having an issue at school, some of the kids will open and be like, ‘oh, that happened to me too, and this is what I did’. So I think there’s definitely value in that. (L-1)

Finally, one of the girls explained that ‘Rose and Thorn’ helped the youth feel like others care. “I liked the talking, just getting to know how people’s days were…so I like the fact that we do ‘Roses and Thorns’ to see how everybody’s week has been. It kind of shows us that we do care” (Y-3). As seen by the above quotations, the girls seemed to thoroughly enjoy the ‘Rose and Thorn’ activity which provided a supportive environment and fostered relational bonds between youth, as well as between youth and leaders.

#### Providing intentional opportunities for leadership

The GJWHF program was designed to end by providing the youth the opportunity to teach a PA-based life skill activity to their younger peers—BGC members between the ages of seven and nine that did not participate in the GJWHF program. This was perceived as being a great success by the youth, as expressed by these two quotations: “I felt like a leader for the kids to help them have fun…felt proud about the game I came up with and it was really good” (Y-4), and “we taught the other kids…that was fun. You learn different things from it…like to help others and to teach other people what I’ve learned” (Y-2). This activity was also reinforced as a success by the leaders, as they recognized that when provided such opportunities the youth step up, are able to teach and take a leadership role. One of the leaders discussed the impact of this activity: “some of the girls had some great teachable moments, like where they brainstormed a quality and then defined it to other younger youth” (LL-Apr. 17, 2012). Furthermore, it was outlined by this leader that the youth seemed to take great initiative when planning and implementing these activities for the younger club members.

The youth took great ownership and pride in teaching their activity, even during the practice run the week before. They were well-spoken in their explanations to the group and showed a lot of confidence, maturity, and put forth a good effort. They did demonstrations and used constructive feedback provided by leaders. (LL-May 9, 2012)

Another leader highlighted during a session debrief that the youth were “very creative and the young girls had a lot of fun participating. One group went as far as bringing treats for the kids and adding it in as part of the obstacle course. They really took great ownership” (LL-May 9, 2012). Finally, one of the program leaders reflected on the overall process of the youth-led end-of-program activity:

For the teaching part, I think it went really well; better than originally expected. They took on a great leadership role within their own groups when they were planning and developing the activities. It gave them their own purpose, like it was on them as to what they were going to choose—everything was left up to them, which was great. I think they really enjoyed having that flexibility…it was all independent and I think they really enjoyed that. (L-5)

#### Having communicative program leaders who supported one another

Over the course of the program, a major success was having a strong supportive network of program leaders who were responsible for implementing the program. As one leader explained, consistent communication was critical to effective implementation:

Open lines of communication. We always sent out an email once a week beforehand and just kind of talked about what we’re going to be doing, does anyone have any questions or suggestions on how we can make this session better…so I think that’s been really good. (L-5)

This helped the planning process over the course of the program to continually keep all leaders involved in what each session would entail. The consistent communication via email also provided opportunities for any of the leaders to deliver feedback or suggestions. Furthermore, in addition to communicating for logistical reasons, leaders communicated with each other for guidance and advice. One leader indicated: “One of the other leaders, she’s been working with the BGC for years and years and she has experience working with youth in camps and things like that, so she’s sort of the person that I turn to” (L-1).

Although the leaders discussed having a supportive network two of the leaders recommended that future years incorporate more training in helping with aspects that arise not directly related to the program curriculum such as dealing with social cliques and issues that the youth may be experiencing outside of the program. One leader shared “We had a debrief with (university supervisor) and at the end of the program, but I think more regular training or informal discussions of the behavioral understanding and how to act on certain behavioral issues that arise.” (L-5) while another leader stated:

More of a structure in training would have been helpful and maybe…knowing how to handle certain situations better. I do find that when there are certain things that people will say, where I’m not really sure how to handle the situation. (L-1)

#### Engaging youth in different types of physical activity

As mentioned, one of the main goals for this program was to provide opportunities for the youth to be physically active. Results found that each session provided sufficient opportunities for youth to engage in a variety of physical activities. From the leaders’ weekly reports of the session implementation, they outlined a number of program achievements with respect to providing an environment supportive of PA. During the program session debriefs, two leaders stated: “all of the activities were successful in getting the girls active while applying a life skill” (LL- Feb. 12, 2012) and “the girls had fun and they were successful in getting their heart rates up and getting them active” (LL-Feb. 23, 2012).

Not only was the program successful in facilitating PA, but also providing opportunities to youth that they might have otherwise not have had the opportunity to do. One leader stated:

A youth-led dance instructor tried a new method of dance with the girls called the GROOVE method. It allowed for a lot of independent movement, and I think the kids really enjoyed it. It gave them a sense of ownership and independence, while allowing for creativity. (LL-Dec. 13, 2011)

Yoga was another activity that many of the youth were able to try for the first time: “Yoga went very well! All the girls were sweating and working hard…they all gave 100% effort and tried very hard (no one gave up). Something new and exciting to do!” (LL-Nov. 1, 2011). Additionally, the youth played lacrosse, went swimming, and skating. The following quotations highlight the youth’s perceptions of the variety of activities: “I really liked the games and how sometimes we would go places—Oh I really liked the swimming activity which was really fun” (Y-1) and “I liked soccer, racquet hockey—oh and huckle buckle. I liked the walking that we did all the way to the river. And swimming–I got to do tricks in the water” (Y-7).

Overall, the youth participants thoroughly enjoyed the PA opportunities they had during the program. This youth discussed how the experience helped her at school: “It got me more active and for some reason it just got me happy and I was just like ‘oh yeah I’m going to have something to do after school!’ and my mom is happy because I’m more active” (Y-3). As mentioned, it is critical to ensure the youth are enjoying themselves during the activities in order to ensure adherence to the program and participation in PA. “We had the girls run a lot, while not really realizing it, and having a lot of fun; you could tell they were all tired at the end but had enjoyed themselves” (LL-Jan. 24, 2012).

### Challenges

As with any program, especially in the first year of implementation, there were challenges. Three main themes emerged which included difficulties with the facility and transportation, recognizing that, at times, some activities were too much like schoolwork, and issues related to and social distractions and cliques.

#### Difficulties with facility and transportation

One challenge that was encountered was the space allocated for the program—it was too small for the group. There was a small craft room for the relational time and awareness talk and GJWHF only had access to the larger gymnasium for the PA time. One leader emphasized this issue:

The room for the life skills, once we were more than 12 girls, it was pretty crammed, so it got loud and sometimes it would be hard to talk over them, so if we could get a bigger room, more private, more isolated. Especially next year, I think we’re estimating to get more girls, so a bigger room would be the ideal. (L-3)

Additionally, as the room in which the girls participated in the life skills activities was right beside the gymnasium, it was often quite loud and distracting having other BGC programming going on at the same time. “If there were people in the gym, the room was right next to the gym and you could hear all the balls thrown and all the kids playing and having fun so it was a bit distracting” (L-3).

As mentioned, another challenge was transportation. A large majority of the participants travelled, with their leader, to the program from a different BGC clubhouse. As a result, over the course of the year, some issues occurred with transportation since the program relied on taxis to transport the youth from clubhouse to clubhouse. The issue of having non-reliable transportation caused serious disruptions in at least two of the program sessions. The leader stated: “Taxis didn't come therefore multiple waves of people caused constant interruptions. Only had 15 minutes to do life skill…a little bit frustrating” (LL-Dec. 6, 2011).

#### Some activities were too much like schoolwork

One challenge was attempting to avoid having the program feel too much like schoolwork. For the life skills activities, the leaders tried to incorporate as many active or discussion-like activities as possible to avoid the feeling of doing schoolwork (e.g., sitting at a desk completing worksheets). However, for some of the life skills activities, a workbook was used to have the youth write down their ideas related to the skill (e.g., thinking about the future, writing down goals for the life skill of goal setting). Therefore, at times, these workbook activities were a challenge as indicated by these two leaders.

If you’re someone who is struggling in school and you come to the club and you want to participate in the program, and you get a workbook that’s full of words you can’t read and you’re sitting at a desk again, I think it kind of re-creates an environment where a lot of our girls aren’t very successful and don’t feel very good about themselves…It’s very school-like. (L-2)

I think having the in-class portion does have its benefits, but it’s just trying to limit the amount of regimented, school-like activities that there are…You have to be adaptable and it might not work out exactly how you planned in the workbook or manual. (L-5)

In addition, many of the activities that the girls chose were similar to what the girls would participate in during a physical education class so the leaders made an extra effort to go beyond what would be done in within the school curriculum.

We tried to plan stuff that you wouldn’t necessarily do in gym class, or if you do soccer or basketball, try and go one step further than what they [the youth] would do in gym class. If they wanted to play a game of bump, play a game of bump, rather than do drills. You could tell they didn’t love it [doing drills] so we kind of left it to be a little bit more unstructured and up to the youth to decide. (L-5)

One of the most important elements the leaders recognized early in the program was that the youth were not as engaged when the PA was regimented like a sport practice so leaders worked to ensure that the activities were not rigid and more enjoyable for the youth.

As long as they’re moving and getting PA, that’s the most important part of it. I guess be on the basketball team if you want to learn the more structured stuff, so that was something that we had to re-adjust—we were like ‘okay, this isn’t working as well as we hoped. We’ll move on and try it a different way’. (L-5)

#### Social distractions and cliques

In line with the above challenge, the leaders struggled with behavioral management of the youth a few times over the course of the program. The youth were from two different clubhouses and the youth who were from the clubhouse in which the program was run were often very distracted by other people and other programs at the club. Therefore, there was an ongoing issue of these youth milling in and out of the sessions at their own leisure. Towards the end of the program this became frustrating not only to the leaders, but also the other youth. While one leader expressed that there was “bickering between certain girls” (LL-Feb. 7, 2012), another leader stated:

In some ways, I think it’s easier for them [girls at home clubhouse] because they have other options, so if they’re feeling shy, they can just go hide in the computer room where the girls [from other clubhouse] don’t have those options. They bus here and this is the program and they’re going to participate in the program. (L-4)

Although not discussed during the interviews, some of the youth who attended the program on a regular basis openly discussed their frustration with the lack of commitment of some of the participants displayed during the program sessions. Related to this, the leaders also occasionally struggled with behavioral challenges due to having all of the girls in one group and so a leader suggested changing this in the future:

Separate them [the youth] into smaller groups…something that we want to work on for next year. Maybe get one or two more leaders…if we do get a big group of 20 girls, split up 10 and 10 and then break those groups smaller. (L-5)

In addition to informal discussions that took place throughout the program, some of the youth took the opportunity to express their concern on their confidential paper indicating elements of the program they did not like: “what I don't like about the GJWHF is that there are no communications between the (name of clubhouse) girls or with the (name of clubhouse) girls and that would be the only reason why I don't really like the program” (AYF-9) and “I think I didn't really talk to the (name of clubhouse) girls that much and I wanted to” (AYF-8). When asked what she did not like about the program, one youth stated: “the (name of clubhouse) girls. It’s not like we’re trying not to talk to them, but I don’t know, we just didn’t bother talking to them as much” (P-10). Lastly, a leader that works at the clubhouse where the program was held had built a strong relationship with the youth from this clubhouse over many years and tried to explain why this issue might have occurred:

For our girls, the ones at (name of clubhouse), I think they actually were a little bit uncomfortable with the idea that there were new girls coming to the centre that they didn’t know and that was kind of like—you know, girls that age, they kind of have their friends and it’s a little bit uncomfortable to meet new people. (L-4)

## Discussion

This evaluation represents an important step in responding to calls for increased evaluation in community-based programs (Salmon et al. 2007) and aids in understanding the processes in which PA-based PYD programs can be effectively designed and implemented. The aim of GJWHF was the program was to engage youth in PA and facilitate life skill development which is believed to help youth develop into healthy adults (Damon 2004; Holt and Jones 2008). According to reports by leaders and participants, the overall program structure worked well with effective relational time, opportunities to develop life skills and time to be physically active. This supports the work of Ward and Parker (2013) which indicated that when the goals of the program align with the delivered activities, resulting in a supportive atmosphere, then healthy development is most likely to be fostered. In addition, the data from the Leader Logbook indicated that GJWHF was implemented, for the most part, as it was intended and that the leaders were successful in achieving the three program goals which were to provide youth with opportunities to be physically active, facilitate the development of life skills, and enable opportunities for the female youth to have voice in the program.

An important outcome that emerged from the GJWHF program was the facilitation of positive relationships between the youth and leaders, as results from this study indicated that the program was successful in providing an environment in which the girls felt comfortable and trusted by their leaders. More specifically, the interview data indicated that these relationships and trust appear to have been facilitated with the use of the ‘Rose and Thorn’ activity at the beginning of each session. It is possible that by providing an intentional time at the beginning of each session for the youth to share both positive and negative moments of the past week with each other allowed them to learn more about each other, listen to each other, and support each other helped which facilitated a sense of trust. This finding is consistent with previous research in which Markowitz (2012) identified that two main ingredients can help female youth increase their self-esteem are structured activities that focus on skill-building and providing a supportive environment. Moreover, research has identified the importance of structured afterschool programs that provide youth with access to caring non-familial adults (Armour et al. 2013; Eccles et al. 2003).

Within the current literature on the TPSR model, suggestions for how to facilitate relational time are quite vague in that it is suggested to check in with the youth (Hellison, 2011). For example, Walsh (2008) suggested having “informal conversations” (p. 62) with youth, such as asking how their day was going or how they were doing in school. From the positive feedback received by youth and leaders within GJWHF, it would be recommended to include a more structured activity such as ‘Rose and Thorn’ or a similar activity to optimize relational time in each program session.

The results of this study also suggest that when youth are provided the opportunity to lead, in particular by teaching younger peers, they may be empowered by being able to take ownership of different activities. Recent research conducted on youth involvement and PA contexts found that creating opportunities for female youth to share their experiences with others and build relationships with peers may help female youth develop their confidence as leaders (Yoshida et al. 2011). Therefore, it appears that using a combination of strategies such as using the ‘Rose and Thorn’ activity during relational time to foster peer relationships and providing intentional opportunities for youth to be leaders by teaching their younger peers may enhance the effectiveness of PA and life skills programs for female youth.

Researcher flexibility is essential when conducting program evaluations, yet program leader flexibility is also critical when facilitating youth-driven programs (Ozer et al. 2008; Ward and Parker 2013; Wright et al. 2012). Over the course of the year, many modifications had to be made to the program structure in order to adapt to better meet the needs of the participants, such as modifying the physical and life skill activities to ensure that the voice of the youth participants was being integrated. From this study, it was found that this can be accomplished by maintaining continuous and open lines of communications between leaders. The importance of regular meetings with all staff is critical in order to communicate and make adjustments if needed. Results suggested that providing voice to the youth in this way led to the incorporation of a variety of physical activities that the youth enjoyed into the program. Being flexible and making modifications to provide voice for the youth are supported by the work of Denner et al. (2005). They discussed that female youth can be empowered when they have the opportunity to build positive relationships with adults and that these relationships are best fostered when a space is created where the youth can have voice. In addition, Wright et al. (2012) stated: “this stresses the need for us, as the leaders, to be flexible, always listening, learning, and adapting the program to best fit the participants’ needs in order to maximize the benefits that can be attained for these girls” (p. 20).

While this study was successful in understanding the processes of implementing the GJWHF program, there were some limitations within the study. First, there is always the potential of self-monitoring when participants know a program is being evaluated. Second, the majority of the data analyzed was based on self-report data; however, using the Leader Logbook helped to align what was discussed by the participants. Additionally, having the program leader conduct the youth interviews could have had the potential for youth social desirability; however, as stated earlier, this is not something the authors felt was an issue and some researchers believe that having leaders interview youth may be an effective as the youth are apt to open up and discuss their experiences with someone with whom they are familiar (Heath et al. 2009).

### Practical implications

Hirsch (2005) indicated that implementation problems are universal, and are not unique to youth programs, but occur in almost every place society works with complex social issues. Therefore, programmers and researchers should come to expect implementation problems even when employing a structured program under highly supportive conditions and sufficient resources. As noted above, challenges occurred during the program, yet the leaders and programmers have learned from these and have incorporated these lessons learned into the second year of the GJWHF program that started in September 2012. More importantly, these lessons learned also serve as recommendations for other programmers or researchers working with community organizations to develop and implement PA-based life skills programs for youth. First, it is critical to have a suitable space for the program. For the second year of GJWHF it was decided to change the day of implementation which resulted in the program being run on a night that the BGC location is closed for all other activities. This decision helped eliminate distractions that were pertinent in the first year of program implementation. Additionally, this change allowed for increased program space which enabled leaders to split the group of youth into two or three smaller groups. As a result, the leaders were able to work in small groups (five to six youth per group) with two leaders for each group. Breaking the larger group of youth into smaller groups was recommended as this strategy can also help minimize difficulties related to behavioral management. The challenges related to social cliques primarily arose due to a lack of consistent participation by some girls which caused friction with girls who did attend the GJWHF program regularly. This is consistent from research conducted within BGCs in the United States that indicated much of staff time is “spent addressing interpersonal conflicts among youth and preventing confrontations” (Hirsch 2005 p. 37). This was also noted within the Smart Girls program, where peer group dynamics were an ongoing issue within the program, particularly as they were female youth between the ages of 10 and 15 (Hirsch 2005). Therefore, for year two of the program it was requested that the participants decided together on the level of commitment they wanted to make, as well as decide on a consequence for youth who did not keep that commitment.

With regards to transportation, instead of relying on taxis and clubhouse vans, for the second year of the program the BGC provided a bus that allowed for all of the youth to be transported to and from the program together. Having reliable and secure transportation helped avoid program interruptions and brought the youth together prior to beginning of the program session. It should be noted that while it is recognized that there are no youth quotations related to this theme, it was because the researcher did not ask the youth about this issue specifically; however, this theme was very apparent to the leaders.

Lastly, within this study, it was requested by the leaders to have more in-depth training prior to and during program implementation. Although the requested training was for issues that were not directly related to the program curriculum it is important that leaders feel competent to be able to deal effectively with all aspects of the program and not just the skills and activities planned for the program sessions. This is consistent with previous TPSR process evaluations that struggled with adequate training for their leaders as “it is not realistic to expect that the Youth Leaders can learn to become effective TPSR leaders through mere observation or through a 10 minute discussion before or after the first session” (Wright et al. 2012 p. 20). Therefore, more training prior, particularly related to managing social cliques and discussing ongoing personal challenges of the youth, may be particularly important for leaders working with all-female programming. In the second year of the GJWHF the program leaders met every two weeks with the professor overseeing this project to discuss successes and ongoing concerns and planned for the two upcoming sessions. This enabled the leaders to feel better prepared and allowed for more program flexibility based on the youth’s choice of sport or PA and enabled a better integration of the life skill within the chosen activity.

## Conclusion

This paper provided a detailed description of the first year of implementation of the GJWHF program. Such detailed descriptions and process evaluations have been absent in much of the PYD literature (Hodge et al. 2013). As Durlak and DuPre (2008) argued, collecting process data is an essential part of program evaluation. The qualitative nature of this study allowed for an in-depth understanding of the successes and challenges of implementing GJWHF and these findings may be helpful to others involved in implementing programs focused on girls’ physical activity and development. For example, this study showed that relational time, providing voice and leadership opportunities along with a variety of physical activities are important as they were perceived as having a positive impact on the participants. Having ongoing communication between leaders was noted by the leaders as imperative to successful program implementation. Further, practitioners and researchers should ensure that youth can access the program easily by providing transportation, avoiding activities that are academic in nature, and working with the youth to commit to the program and foster relationships to avoid potential issues with behavioral management. In sum, we hope that this research can help improve the implementation of programs designed to enhance the health and well-being of female youth.

## Abbreviations

PYD: Positive youth development
PA: Physical activity
BGC: Boys and Girls Club
GJWHF: Girls Just Wanna Have Fun.

## References

[CR1] Armour K, Sandford R, Duncombe R (2013). Positive youth development and physical activity/sport interventions: mechanisms leading to sustained impact. Phys Educ Sport Pedagog.

[CR2] (2013). Smart Girls.

[CR3] Braun V, Clarke V (2006). Using thematic analysis in psychology. Qual Res Psychol.

[CR4] (2011). Don’t let this be the most physical activity our kids get after school. The active healthy kids Canada 2011 report card on physical activity for children and youth.

[CR5] Catalano RF, Berglund ML, Ryan JA, Lonczak HS, Hawkins JD (2002). Positive youth development in the United States: research findings on evaluations of positive youth development programs. Prevention Treatment.

[CR6] Coakley J (2011). Youth sports: What counts as “Positive Development?”. J Sport Soc Issues.

[CR7] Cooky C (2009). “Girls just aren’t interested”: The social construction of interest in girls’ sport. Sociol Perspect.

[CR8] Creswell JW (2013). Research design: Qualitative, quantitative, and mixed methods approaches.

[CR9] Damon W (2004). What is positive youth development?. Ann Am Acad Polit Soc Sci.

[CR10] Danish SJ (1997). Going for the goal: a life skills program for adolescents. Issues in Childrens and Families Lives.

[CR11] Danish SJ, Forneris T, Hodge K, Heke I (2004). Enhancing youth development through sport. World Leisure J.

[CR12] DeBate RD, Pettee Gabriel K, Zwald M, Huberty J, Zhang Y (2009). Changes in psychosocial factors and physical activity frequency among third- to eighth-grade girls who participated in a developmentally focused youth sport program: a preliminary study. J Sch Health.

[CR13] Denner J, Meyer B, Bean S (2005). Young Women’s Leadership Alliance: youth–adult partnerships in an all‒female after‒school program. J Community Psychol.

[CR14] Dobbins M, De Corby K, Robeson P, Husson H, Tirilis D (2009). School-based physical activity programs for promoting physical activity and fitness in children and adolescents aged 6–18. Cochrane Database Syst Rev.

[CR15] Durlak JA, DuPre EP (2008). Implementation matters: a review of research on the influence of implementation on program outcomes and the factors affecting implementation. Am J Community Psychol.

[CR16] Eccles JS, Barber BL, Stone M, Hunt J (2003). Extracurricular activities and adolescent development. J Soc Issues.

[CR17] Finlay L (2014). Engaging phenomenological analysis. Qual Res Psychol.

[CR18] Forneris T, Bean CN, Snowden M, Fortier M (2013). Using youth-driven programs to encourage physical activity in adolescent girls: A preliminary study. PHEnex J.

[CR19] Futris TG, Sutton TE, Richardson EW (2013). An evaluation of the Relationship Smarts Plus program on adolescents in Georgia. J Hum Sci Extension.

[CR20] (2013). Girls Inc. Sporting Chance.

[CR21] Gould D, Carson S (2008). Life skills development through sport: current status and future directions. Int Rev Sport Exerc Psychol.

[CR22] Heath S, Brooks R, Cleaver E, Ireland E (2009). Researching young people’s lives.

[CR23] Hellison DR (1995). Teaching responsibility through physical activity.

[CR24] Hellison DR (2011). Teaching personal and social responsibility through physical activity.

[CR25] Hellison D, Walsh D (2002). Responsibility-based youth programs evaluation: investigating the investigations. Quest.

[CR26] Hellison D, Martinek T, Walsh D, Holt N (2008). Sport and responsible leadership among youth. Positive youth development through sport.

[CR27] Hirsch BJ (2005). Structured Programs: The Implementation of Smart Girls.

[CR28] Hodge K, Danish S, Martin J (2013). Developing a conceptual framework for life skills interventions. Couns Psychol.

[CR29] Holt NL (2008). Positive youth development through sport.

[CR30] Holt NL, Jones MI (2008). Future directions for positive youth development and sport research. Positive youth development through sport.

[CR31] Kelly P, Matthews A, Foster C (2012). Young and physically active: a blueprint for making physical activity appealing for youth.

[CR32] Linnan L, Steckler A (2002). Process evaluation for public health interventions and research.

[CR33] Markowitz E (2012). Exploring self-esteem in a girls’ sports program: Competencies and connections create change. Afterschool Matters.

[CR34] Martin JJ, Waldron JJ, McCabe A, Choi YS (2009). The impact of “Girls on the Run” on self-concept and fat attitudes. J Clin Sport Psychol.

[CR35] Martinek T, Schilling T, Johnson D (2001). Transferring personal and social responsibility of underserved youth to the classroom. Urban Rev.

[CR36] Martinek T, Schilling T, Hellison D (2006). The development of compassionate and caring leadership among adolescents. Phys Educ Sport Pedagog.

[CR37] Maxwell JA (2005). Qualitative research design: An interactive approach.

[CR38] Nakkash RT, Alaouie H, Haddad P, El Hajj T, Salem H, Mahfoud Z, Afifi RA (2012). Process evaluation of a community-based mental health promotion intervention for refugee children. Health Educ Res.

[CR39] Ozer EJ, Cantor JP, Cruz GW, Fox B, Hubbard E, Moret L (2008). The diffusion of youth-led participatory research in urban schools: the role of the prevention support system in implementation and sustainability. Am J Community Psychol.

[CR40] Peters DH, Tran NT, Adam T (2013). Implementation research in health: a practical guide.

[CR41] Petitpas AJ, Cornelius AE, Van Raalte JL, Jones T (2005). A framework for planning youth sport programs that foster psychosocial development. Sport Psychol.

[CR42] Pollio HR, Henley TB, Thompson CJ (1997). The phenomenology of everyday life.

[CR43] Rauscher L, Kauer K, Wilson BDM (2013). Organizational and interactional influences on preadolescent girls’ body image in Los Angeles. Gend Soc.

[CR44] Salmon J, Booth ML, Phongsavan P, Murphy N, Timperio A (2007). Promoting physical activity participation among children and adolescents. Epidemiol Rev.

[CR45] Saunders RP, Evans MH, Joshi P (2005). Developing a process-evaluation plan for assessing health promotion program implementation: a how-to guide. Health Promot Pract.

[CR46] Shek DT, Sun RC (2012). Process evaluation of a positive youth development course in a university setting in Hong Kong. Int J Disabil Hum Dev.

[CR47] Stone EJ, McKenzie TL, Welk GJ, Booth ML (1998). Effects of physical activity interventions in youth: review and synthesis. Am J Prev Med.

[CR48] Theokas C, Almerigi JB, Lerner RM, Dowling EM, Benson PL, Scales PC, von Eye A (2005). Conceptualizing and modeling individual and ecological asset components of thriving in early adolescence. J Early Adolesc.

[CR49] Thomas H, Ciliska D, Micucci S, Wilson-Abra J, Dobbins M (2004). Effectiveness of physical activity enhancement and obesity prevention programs in children and youth.

[CR50] Waldron JJ (2007). Influence of involvement in the girls on track program on early adolescent girls’ self-perceptions. Res Q Exerc Sport.

[CR51] Walsh D (2008). Strangers in a strange land: Using an activity course to teach an alternative curriculum model. J Phys Edu, Rec &Dance.

[CR52] Walsh DS, Ozaeta J, Wright PM (2010). Transference of responsibility model goals to the school environment: Exploring the impact of a coaching club program. Phys Educ Sport Pedagog.

[CR53] Ward S, Parker M (2013). The voice of youth: atmosphere in positive youth development program. Phys Educ Sport Pedagog.

[CR54] Weiss MR, Wiese-Bjornstal DM (2009). Promoting positive youth development through physical activity. President's Council on Physical Fitness and Sports Research Digest.

[CR55] Whitley MA, Forneris T, Barker B (2014). The reality of evaluating community-based sport and physical activity programs to enhance the development of underserved youth: Challenges and potential strategies. Quest.

[CR56] Windsor RA, Baranowski T, Clark N, Cutter G (1984). Evaluation of Health Promotion and Education Programs.

[CR57] Wright PM, Stockton M, Hays NL, Coulter J (2008). The personal-social responsibility model: Exploring a novel approach to promoting gender equity and increasing relevance for adolescent females in physical education. Progress in Exercise and Women’s Health Research.

[CR58] Wright EM, Whitley MA, Sabolboro G (2012). Conducting a TPSR program for an undeserved girls’ summer camp. Agora PE Sport.

[CR59] Yin RK (2009). Case study research: Design and methods.

[CR60] Yoshida SC, Craypo L, Samuels SE (2011). Engaging youth in improving their food and physical activity environments. J Adolescent Health.

